# Distribution and metabolism of daidzein and its benzene sulfonates *in vivo* (in mice) based on MALDI-TOF MSI

**DOI:** 10.3389/fphar.2022.918087

**Published:** 2022-08-10

**Authors:** Yanxiao Jiao, Xueqin Li, Yao Tang, You Peng, Guisen Chen, Xin Wang, Long Yan, Huihui Liu, Zongxiu Nie

**Affiliations:** ^1^ College of Chemistry and Chemical Engineering, Jiangxi Province Engineering Research Center of Ecological Chemical Industry, Jiujiang University, Jiujiang, China; ^2^ State Key Laboratory of Food Nutrition and Safety, College of Food Science and Engineering, Tianjin University of Science and Technology, Tianjin, China; ^3^ Key Laboratory of Analytical Chemistry for Living Biosystems, Institute of Chemistry Chinese Academy of Sciences, Beijing, China

**Keywords:** daidzein, benzenesulfonate derivatives, metabolic distribution *in vivo*, MALDI-TOF MSI, sulfonation

## Abstract

Daidzein (D1) has been proved to be of great benefit to human health. More and more attention was paid to the metabolic process of D1. Most studies focused on the metabolites of D1 and analogs were determined through the excretion of animals and humans by traditional HPLC-MS, while their *in situ* distribution and metabolism in organs *in vivo* has not been reported. In our group, novel daidzein sulfonate derivatives were synthesized and confirmed to have excellent pharmaceutical properties. They exhibited good anti-inflammatory, inhibitory activities on human vascular smooth muscle cell proliferation and other bioactivities. Compared with traditional analytical methods, matrix-assisted laser desorption ionization time-of-flight mass spectrometry imaging (MALDI-TOF MSI) can directly analyze the distribution of compounds in tissues and organs. In this study, we investigate the *in situ* distribution and metabolism of D1 and its derivatives (DD2, DD3) in the organs of mice based on MALDI-TOF MSI for the first time. Trace prototype compounds were detected in the plasma 4 h after the intravenous injection of D1, DD2, and DD3. Seven phase I metabolites and seven phase II metabolites were detected. D1 sulfates were found in the plasma and in organs except the heart. The presence of D1 and DD3 monosulfates in the brain indicated that they could penetrate the blood–brain barrier. DD2 and DD3 could be hydrolyzed into D1 and their metabolic pathways were similar to those of D1. In addition, a ligand-receptor docking of D1 and DD2 with mitogen-activated protein kinase 8 (JNK1) was performed because of their significant anti-inflammatory activities through the JNK signaling pathway. It showed that the binding energy of DD2 with JNK1 was obviously lower than that of D1 which was consistent with their anti-inflammatory activities. It provided a theoretical basis for further validation of their anti-inflammatory mechanism at the protein level. In summary, the research will provide beneficial guidance for further pharmacological, toxicological studies and the clinical-use research of these compounds.

## 1 Introduction

**FIGURE 1:**
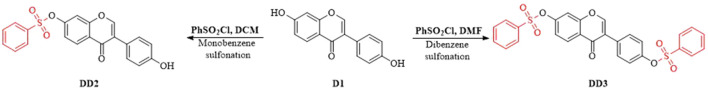
Synthesis of D1 benzene sulfonate derivatives.

Daidzein (4′, 7-dihydroxyisoflavone, D1) is considered to be one of the major active components in soybean. Accumulating evidence indicates that D1 has great health benefits (Zaheer and Humayoun, 2017) and diverse biological activities, including not only anti-inflammatory (Peng et al., 2017), anti-oxidant (anti-aging) (Garbetta et al., 2014), anti-atherosclerotic (Roghani et al., 2013; Karlíčková et al., 2016), and hyperglycemia lowering effects (Huang et al., 2004; Cheong et al., 2014), but also anti-cancer (Bao et al., 2014), anti-cardiovascular diseases (Li and Zhang, 2017), and reduction of the menopausal syndrome in women (Chen and Chen, 2021). However, the two polar hydroxyl groups of D1 result in its weak lipophilicity, and the high lattice energy arising from the intermolecular hydrogen bonds of the hydroxyls results in its weak hydrophility. Thus, its low solubility leads to reduced bioavailability and biological activities. So far, D1 has not been widely and effectively used in clinical studies. To improve the pharmacological action of D1, our group designed and synthesized daidzein sulfonate derivatives based on the principle of pharmacokinetic group transformation and studied their pharmaceutical properties and bioactivities (Peng et al., 2009) (Figure 1). Their pharmaceutical properties were optimized compared to D1 (Peng and Deng, 2011). It was observed that the water solubility and fat solubility of the sulfonate derivatives were greatly improved (Jiao et al., 2021). The results showed that they had some anti-cancer activity (Peng et al., 2009) and good inhibition of human vascular smooth muscle cell (HVSMC) proliferation and migration (Zhao et al., 2012). Our previous results showed that the anti-inflammatory property of D1 sulfonate derivatives DD2 and DD3 were much better than that of D1 *in vitro* (Peng et al., 2017). In this study, we performed an *in situ* distribution and metabolism research of D1, DD2, and DD3 *in vivo* to further study their pharmacology, toxicology, and clinical use.

**SCHEME 1:**
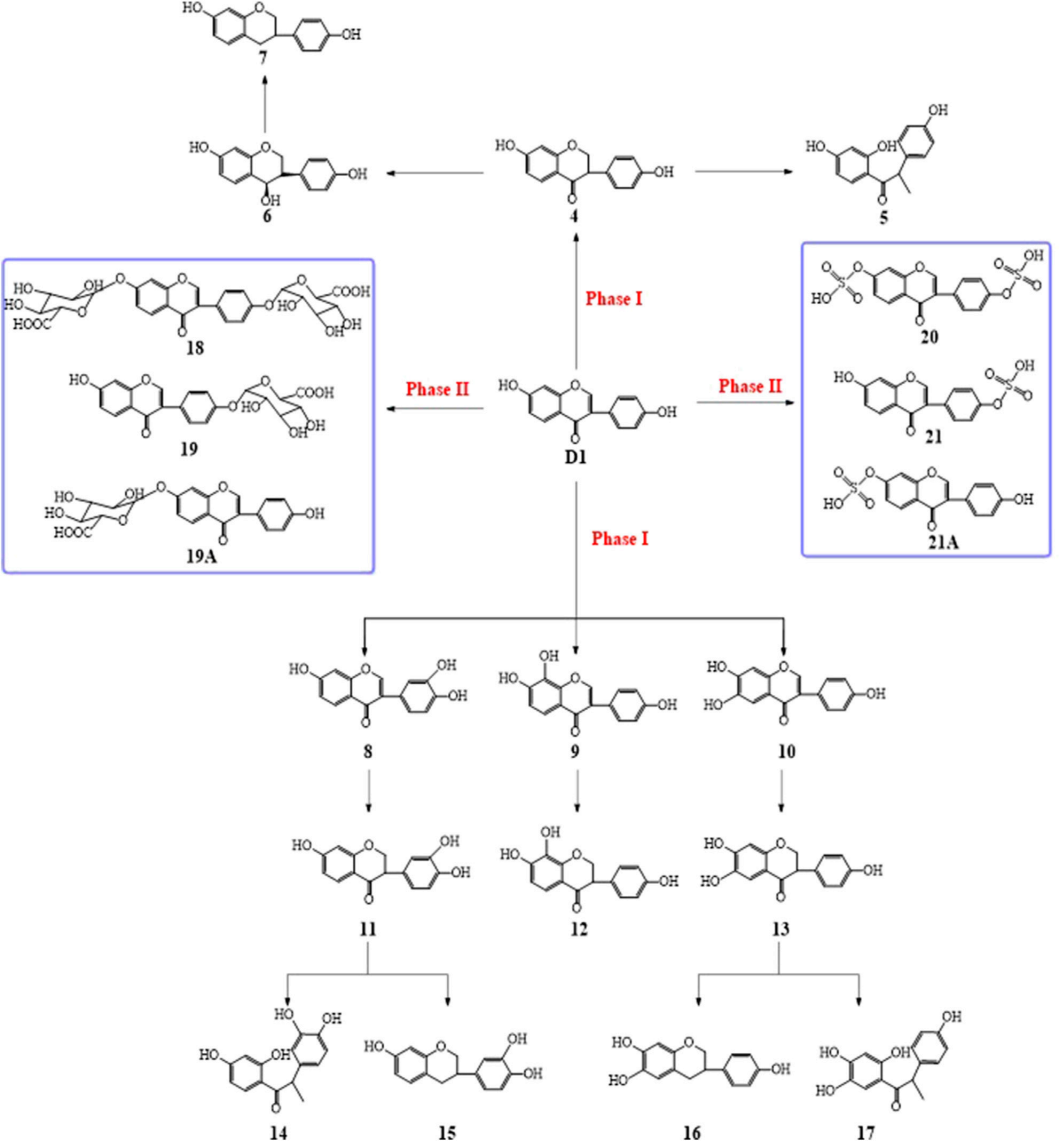
Possible metabolic pathways of daidzein *in vivo*.

In order to evaluate the potential risks and health benefits of D1 and to better utilize the health benefits of D1, it is important to understand the metabolic processes and metabolites of D1. At present, the metabolic process of D1 and analogs *in vivo* was studied via the determination of the metabolites in the plasma, in excretion (urine or feces) or *in vitro*, such as microsomes, bacteria, and cells. Kulling (Kulling et al., 2000; Kulling et al., 2001; Kulling et al., 2002) *et al.* identified nine oxidation metabolites of D1 in human urine using high-performance liquid chromatography (HPLC) and gas chromatography–mass spectrometry (GC–MS), including four monohydroxylated, four dihydroxylated, and one trihydroxylated metabolites, most of which were reported for the first time. At the same time, they used the metabolites of D1 in human and rat liver microsomes as standards to determine the structure of these oxidative metabolites. Adlercreutz’s (Adlercreutz et al., 1995; Adlercreutz et al., 1999; Heinonen et al., 2003) group proposed the metabolic pathway of D1 based on the identification of metabolites in the urine of volunteers who consumed soybean. The metabolic pathway of D1 is shown in Scheme 1 (Adlercreutz et al., 1999). The metabolites of D1 in urine were analyzed by GC–MS including phase I (oxidation, reduction) and phase II (glucuronidation, sulfonation) metabolisms. They also further identified the structure of D1 metabolites in human urine obtained from incubating D1 with human fecal flora (Heinonen et al., 2004). Shelnutt (Shelnutt et al., 2000; Shelnutt et al., 2002) and others reported the pharmacokinetics of glucuronide and sulfate conjugates of D1 in human urine and plasma, respectively. The results showed that there were significant differences in the pharmacokinetics of D1 sulfates and glucuronide conjugates in the urine and plasma. D1 was excreted mainly in the form of sulfates and glucuronide conjugates into urine. However, the concentrations of D1 sulfates and glucuronide conjugates were observably lower in the plasma than in urine. Zhang (Zhang et al., 2003) *et al.* analyzed female urine and plasma samples by HPLC. It was found that one-fifth of D1 will enter the urine through circulation and glucuronides were the main metabolites. Redmon (Redmon et al., 2016) *et al.* investigated metabolites in urine samples from cats and dogs and liver microsomes from cats compared with microsomes from 12 other species. The results showed that sulfation was probably the major metabolic pathway, while glucuronidation was a minor pathway for D1 metabolism in cats. It demonstrated that there were species differences in the metabolism of D1 in different animal species. Zhao (Zhao et al., 2018) *et al.* developed a rapid strategy to identify D1 derivatives in the urine and plasma of rats based on UHPLC-LTQ-Orbitrap MS combined data-mining methods. A total of 59 metabolites including D1 were identified, of which only two could be found in the plasma. Hanioka (Hanioka et al., 2018) studied the regioselective glucuronidation of D1 at 7-and 4′- hydroxyl groups in human, monkey, rat, and mouse liver and intestinal microsomes. The results showed that the metabolic ability of UDP-glucuronosyltransferase toward D1 in the liver microsomes and the intestinal microsomes was significantly different in humans, monkeys, rats, and mice. Kim (Kim et al., 2008), Wang (Wang et al., 2021), and Soukup (Soukup et al., 2021) studied the metabolites of isoflavones in intestinal bacteria respectively. Kim’s research found that D1 could be directly metabolized into dihydrodaidzein and equol by intestinal microflora before absorption. Wang *et al.* established the UPLC–Q-TOF/MS technique for the identification of daidzin and its metabolites in human intestinal bacteria. It was found that D1 was methoxylated to the corresponding metabolite methoxylated D1 and further hydroxylated to the probable metabolite hydroxylated D1 by intestinal bacteria. Soukup investigated the metabolisms of D1 and genistein by the human gut bacterial strains “*Hugonella massiliensis*” DSM 101782T and *Senegalimassilia faecalis* KGMB 04484T. It was considered that different strains played different roles in the metabolism of D1. Toro-Funes (Toro-Funes et al., 2015) and others described the metabolism of D1 in human endothelial (HUVEC), liver (HepG2), and intestinal epithelial cells (Caco-2 monolayer). Their results indicated that D1 was metabolized into methoxy-D1-glucuronides in HUVEC, D1 glucuronide conjugates in HepG2 cells and D1 sulfate conjugates in Caco-2 cells. Our group evaluated the metabolisms of two novel D1 napsylates in human aortic vascular smooth muscle cells (HAVSMCs) (Jiao et al., 2021). The D1 napsylates were hydrolyzed into D1 under the action of intracellular hydrolase ester hydrolysis or ether hydrolysis. The glucuronidation and sulphation occurred simultaneously, and they are competitive reactions depending on the concentration of the prototypes (Su, 2008). The aforementioned reports mainly focused on the investigation of the metabolites of D1 and its derivatives in two aspects: 1) The metabolic processes of D1 and analogs *in vivo* were presumed by investigating their metabolites in the plasma or in excretion (urine or feces) of humans or animals. 2) The metabolites of D1 and analogs in microsomes, bacteria, and cells were determined to explore their metabolic process *in vitro*. However, these studies cannot directly describe the metabolism and distribution of D1 and its derivatives in organs and tissues *in vivo*.

Though a wealth of information has been obtained from previous studies on D1 metabolites, these traditional mass spectrometry methods can not directly describe the distribution and metabolism of D1 in organs and tissues *in vivo*. Relative to traditional HPLC-MS, matrix-assisted laser desorption ionization mass spectrometry imaging (MALDI MSI), emerging in recent years and possessing unique features, namely being free of isotope labeling, high sensitivity, simple sample processing, high throughout, and molecule-specificity, has the capacity for *in situ* localizing a wide range of biomolecules simultaneously from a tissue specimen in one single run (Mas et al., 2020; Stopka and Vertes, 2020; Sun et al., 2020; Xie et al., 2021). In this study, we determined the distribution and metabolism of D1 and its derivatives in the plasma and organs of mice based on MALDI-TOF MSI. Since these compounds could exhibit significant anti-inflammatory activities through the JNK signaling pathway in our previous work (Peng et al., 2017), we also performed molecular docking of D1and DD2 with mitogen-activated protein kinase 8 (JNK1) which was the key protease in the JNK signaling pathway.

## 2 Materials and methods

### 2.1 Chemicals and reagents

D1 (purity >98%) was purchased from Shanxi Huike Plant Development Co. Ltd. (Xi’an, Shanxi, China); 2-cyano-3-(4-hydroxyphenyl)-2-propenoic acid (CCA), N-(1-naphthyl)ethylenediamine dihydrochloride (NEDC), and 2,5-dihydroxybenzoic acid (DHB) for matrix preparation; dimethyl sulfoxide (DMSO), and PEG-400 for the dissolution of drugs; heparin sodium for preventing the coagulation of blood, and chloral hydrate for anesthesia were purchased from Sigma-Aldrich (St Louis, MO, United States). DD2 and DD3 were synthesized in our lab.

### 2.2 Synthesis and physicochemical properties

As described in Ref. 9, benzenesulfonyl chloride (0.48 mmol, 0.0847 g) was added drop-wise to the mixture of D1 (0.4 mmol, 0.1016 g) and potassium *t*-butoxide (0.01 g) in dichloromethane (10 ml) at −20°C under argon protection in 15 min. The mixture continued to react at −20°C for 2.5 h. The reaction solution was filtered, concentrated, and purified by column chromatography to obtain DD2 (0.133 g, 80.9% yield). Similarly, benzenesulfonyl chloride was added drop-wise to the mixture of D1 and potassium *t*-butoxide in *N*, *N*-dimethylformamide at 220°C under argon protection in 15 min. The mixture was stirred for 2.5 h to afford DD3 with 99 % yield (Peng et al., 2009). The crystal structure of DD2 was determined and the crystallographic data are published in Ref. 35. We developed a HPLC method for the determination of their solubilities, lipid–water partition coefficient, and hydrolysis kinetics *in vitro* as reported in Ref. 10. ChemAxon 5.2.1.0 was used to were calculated on log*P*, pka, polar surface area, H-bond, etc., of the compounds. The results showed that the lipid- and water-soluble properties of DD2 and DD3 were significantly increased relative to D1 (Peng and Deng, 2011).

### 2.3 Pharmaceutical activities

Various biological activities of the derivatives were determined in our lab. The anti-cancer activities of D1, DD2, and DD3 were examined via detecting their cytotoxic activities against human leukemia cell line HL-60 and human lung cancer line A-549 (Peng et al., 2009). The results in reference 9 suggest that the inhibition of HL-60 by DD2 and DD3 was higher than that of D1. We evaluated the inhibitory activities of D1 and its derivatives against human vascular smooth muscle cell (HVSMC) proliferation and migration by measuring their absorption using an HPLC method (Zhao et al., 2012). The results showed that DD2 and DD3 effectively regulated cell migration probably because of their significant absorption and metabolism by HVSMCs in literature 12. The anti-inflammatory and anti-oxidative activities of D1, DD2, and DD3 were examined by using an *in vitro* model of inflammation in Caco-2 epithelial cell lines through ELISA and Western Blot (Peng et al., 2017). DD2 and DD3 showed significantly enhanced anti-inflammatory activities compared with that of D1 as shown in reference 2.

### 2.4 *In situ* metabolism and distribution

#### 2.4.1 Solution preparation

CCA (10 mg) was dissolved in 60% acetonitrile/H_2_O (100 μL) using ultrasound; then, CF_3_COOH (2 μL) was added. NEDC (10 mg) was dissolved by vortexing in 30% aqueous ethanol (1 ml). DHB (10 mg) was dissolved using ultrasound in 50% aqueous acetonitrile; then, CF_3_COOH (2 μL) was added. All solutions were stored at 4°C.

D1 or its derivatives (10 mg) was dissolved in dimethylsulfoxide (0.1–0.2 ml) using ultrasound and diluted to 2.0 ml with PEG-400 to prepare a concentration of 5 mg/ml solution of the compound, which was then used for intravenous administration. The solution was freshly prepared and filtered with 0.45 μm organic membrane before use.

### 2.5 Preparation of test animals

The Male Kunming mice (20–22 g, aged several weeks) were provided by the Experimental Animal Center of Peking University. Animal experimentation was performed according to the NIH Guide for the Care and Use of Laboratory Animals (National Institute of Health Publication, No. 3040–2, revised 1999, Bethesda, MD, United States) and was approved by the Animal Care and Use Committee of the Chinese Academy of Sciences. Kunming mice were fasted overnight and had access to water freely before administration. The derivatives DD2 and DD3 were injected into the mice via the tail vein in a 25 mg/kg dose, which was the optimal concentration from the intravenous experiment using D1 as the model.

#### 2.5.1 Preparation of plasma samples

After mice were euthanized with chloral hydrate (350 mg/kg, i. p.), the blood samples were obtained via the angular vein and placed in heparinized Eppendorf tubes. The plasma was separated after centrifuging the blood at 5,000 × g for 10 min. A portion (100–200 μL) of the plasma was transferred to a 1.5-ml tube and stored at -20°C. To extract the D1 metabolites, the frozen plasma was thawed and then 20 μL was mixed with 60 μL of acetonitrile and centrifuged to separate the precipitated proteins. A supernate (2 μL) was mixed with the CCA solution (2 μL) and an aliquot (1 μL) of the mixture was deposited on the MALDI steel target.

### 2.6 Tissue dissection

The mice were anesthetized using chloral hydrate (350 mg/kg, i. p.) and were euthanized by cervical dislocation. The heart, lungs, brain, liver, kidneys, and spleen tissues were then removed and snap-frozen in liquid nitrogen. All tissues were stored at -80°C until further use.

### 2.7 Tissue sectioning

The tissues were fixed on the cutting stage atop a drop of saline to avoid signal suppression of the embedding medium. All tissues were sliced to a thickness of 10 μm using a Leica CM1950 cryostat (Leica Microsystems GmbH, Wetzlar, Germany) at -23°C and thaw-mounted onto conductive indium tin oxide (ITO)-coated glass slides, except for the lung tissues, which were then sliced to a thickness of 15 μm. The glass slides were then dried in a vacuum desiccator for approximately 1 h before matrix application.

### 2.8 Evaluation of the matrix candidate

For the MALDI-TOF analysis, the dried-droplet sample preparation method was used as follows: 2 μL plasma sample solution and 2 μL matrix solution were mixed, and 1 μL of the mixture was then pipetted on to the stainless steel target probe, which was dried under a stream of nitrogen gas at room temperature.

### 2.9 MS analysis

The MALDI-TOF MS analysis was performed on a Bruker BIFLEX III mass spectrometer (Bruker Daltonics, Germany) equipped with a nitrogen laser (337 nm wavelength, 3 ns laser pulse duration) with reflection and in the positive ion mode. The laser power was adjusted to be between 0 and 100% to provide a laser pulse energy of 0–100 μJ per pulse. The mass spectra were typically recorded at an accelerating voltage of 19 kV and a reflection voltage of 20 kV, with a laser pulse energy of 80 μJ. Each mass spectrum was acquired as an average of 90 laser shots at a frequency of 1 Hz, unless stated otherwise. A mixture of PEG-200 and PEG-600 was used for positive mode mass calibration.

### 2.10 MS imaging

For MS imaging (MSI), the matrix solution, which was prepared as described previously, was sprayed on the tissue sections mounted onto the ITO-coated glass slides using an automatic matrix sprayer (ImagePrep, Bruker Daltonics) in order to ensure a homogeneous matrix coverage over the entire tissue surface.

An Ultraflextreme MALDI-TOF/TOF mass spectrometer (Bruker Daltonics, Billerica, MA, United States) equipped with a smartbeam Nd:YAG 355 nm laser was utilized for the MALDI-TOF analysis. The laser was fired at a repetition rate of 2,000 Hz, and the analyzer was operated in the positive reflectron mode. The positive-ion mass spectra in the reflectron mode were collected at a pulsed ion extraction time of 80 ns, an accelerating voltage of 20.00 kV, an extraction voltage of 17.90 kV, a lens voltage of 5.85 kV, and a reflector voltage of 21.15 kV. The laser spot size was set at medium focus (-50 μm laser spot diameter), and the laser power was optimized at the start of each run and then fixed for the whole experiment. The MS data were acquired over a mass range of m/z 0–1,000 Da. Mass calibration was performed using external standards prior to data acquisition. For the MSI analysis, the imaging spatial resolution was set to 200 μm for the spleen, liver, and kidney tissues from mice. Each spectrum consisted of 200 laser shots. The regions of interest were manually defined in the imaging software using both the optical image and MSI data image. The MALDI-TOF mass spectra were processed with the total ion current (TIC) normalization, and the signal intensity of each imaging data was represented as the normalized intensity.

### 2.11 Further detailed structural confirmation

MS/MS fragmentations using the laser-induced dissociation (LIFT) technique on the Ultraflextreme MALDI-TOF MS together with Fourier transform ion cyclotron resonance (FTICR) MS as well as Obitrap MS were used for further confirmation of the identified metabolites. According to the literature report (Zhao et al., 2018), the MDF method for screening metabolites of D1 was established to comprehensively extract the related ions in the data. Then, combined with the co-existence of parent ions, the possible metabolites of D1 were systematically screened. Metabolites were identified or predicted by comparing the MS or MS/MS spectra with those of the standard compounds or referring to the database (METLIN, https://metlin.scripps.edu/; MassBank, https://www.massbank.jp/; Human Metabolome Database, https://www.hmdb.ca/; and LIPID MAPS, https://www.lipidmaps.org/).

### 2.12 Molecular docking

AutoDock (Version 4.2.6), PyMOL (Version 2.5.2), MGLTools (Version 1.5.7), and OpenBabel (Version 3.1.1) were taken from the corresponding official websites as open-source softwares (AutoDock, https://autodock.scripps.edu/download-autodock4/; PyMOL, https://pymol.org/2/; MGLTools, http://mgltools.scripps.edu/; and OpenBabel, http://openbabel.org/wiki/Main_Page). A ligand–protein complex of JNK1 (PBD ID: 3pze) was downloaded from the RCSB PDB database and the crystal structure of D1 (ZINC000018847034) was downloaded from the ZINC database. An X-ray crystal structure of DD2 (CCDD: 674,666) was obtained from our previous experiments (Peng et al., 2007).

The raw files of the crystal structures of D1 and DD2 were converted to pdb files by Openbabel (Version 3.1.1). PyMOL (Yuan et al., 2017) and were used to process the protein–ligand complex (PDB ID: 3pze), remove solvents and other small molecules, and save the pdb files of the original ligand (CFK) (Oza et al., 2012) and JNK1 protein. The workflow of AutoDock was as follows. The first step was preparation of the protein and ligand files. Ligand (CFK, D1, DD2) pdb files were repaired by defining the torsions and the JNK1 protein was repaired by adding hydrogen and computing Gasteiger charges in the AutoDock software. The second step was the AutoGrid calculation. One grid box was used to perform the docking calculations, and its dimensions were set to 60 × 60 × 60 Å^3^, grid center was set to X: 15.684, Y: 15.708, Z:23.434, and the spacing was set to 0.375 Å for all systems. The third step was setting the docking parameters. The number of genetic algorithm runs was set to 200 and other settings were chosen as the defaults in the docking process. Finally, molecular docking was performed to obtain the molecular binding energy of the protein with ligand.

## 3 Results

DD2 and DD3 were designed and synthesized based on the principle of pharmacophore transformation by introducing the sulfonate ester group into D1 to improve the amphiphilicity (hydrophilicity and lipophilicity) (Peng et al., 2009). The results were shown as Part 4 in the supporting file. It was found that lipid solubility and water solubility of DD2and DD3 was significantly improved relative to D1 (Peng and Deng, 2011). The preliminary bioassay of DD2 and DD3 showed that they had some anti-cancer activity, good inhibition of human vascular smooth muscle cell (HVSMC) proliferation and migration, and a significant anti-inflammatory activity (Peng et al., 2009; Zhao et al., 2012) (Part 3 in the supporting file). The results showed that both of the derivatives were effective for HL-60 but not for A-549 (Peng et al., 2009). And, the derivatives exhibited a certain degree of improvement in the inhibition rate against HL-60 than D1 (Peng et al., 2009). When their concentration was 100 μM, the inhibition rates of DD2 and DD3 on HVMSC proliferation were 85 and 73.75, and their inhibition rates on HVMSC migration were 81.17 and 71.43, respectively (Zhao et al., 2012). The results of the anti-inflammatory experiments showed that the anti-inflammatory activity of DD2 and DD3 was 100–10,000 times higher than that of D1 in TNF-α-stimulated Caco-2 cells (Peng et al., 2017) (Part 3 in the supporting file). The anti-inflammatory mechanism of D1, DD2, and DD3 was preliminarily studied via Western blot (WB), which showed that D1 and its derivatives down-regulated the phosphorylation of JNK through the JNK (NH_2_ terminal kinase)/AP-1 (activator protein-1) pathway (Peng et al., 2017).

### 3.1 Evaluation of matrix candidate

The interplay between the wavelength of the laser and the absorption profile of the matrix is a crucial factor in MALDI-TOF MSI. It was showed that the best analytical results were obtained when the laser wavelength matched well with the UV absorption band of the matrix in the solid state. The dried-droplet method of sample application was initially used to assess the commonly used MALDI matrices including CCA, NEDC, and DHB (Supplementary Figure S1). The results suggested that CCA had a relatively strong absorption at the applied laser wavelength, meeting one of the basic requirements of being a good MALDI matrix. The MALDI mass spectra of CCA were acquired in positive ion modes. The positive ion spectrum exhibited a prominent peak at m/z 172.013 (100%), arising from [M−H_2_O + H]^+^, accompanied by a peak at m/z 146.018, corresponding to [M−H_2_O−CN + H]^+^, which was not so abundant (Supplementary Figure S1F).

The positive ion spectrum of D1 exhibited a prominent peak at m/z 276.835 (100%), arising from [M + Na]^+^ (Supplementary Figure S1C). The intensity of the peaks at m/z 145.908 and m/z 171.868 from [M_CCA_−H_2_O−CN + H]^+^ and [M_CCA_−H_2_O−CN + H]^+^ decreased significantly, while the signal of the peak at m/z 254.902 from [M_D1_+H]^+^ was strong in the spectrum of D1 with CCA (Supplementary Figure S1D). The results indicated that the mass spectrum of CCA was very clean with few matrix-derived background signals, especially at m/z values <500. The ratio of the (CCA + D1) peak intensity and the D1 peak intensity was 3.62, and the ratio of (DHB + D1) peak intensity and D1 peak intensity was 2.79 (Supplementary Table S1). However, D1 peaks could not be detected by MS from a mixture of NEDC and D1 (Supplementary Figure S1B), and CCA showed fewer background signal interferences than DHB. Furthermore, CCA could detect all the D1, DD2, and DD3 with high intensities when compared with DHB, under optimized conditions for each matrix. In a word, CCA was the most suitable matrix in this experiment.

### 3.2 Analysis of metabolites in mice plasma

**FIGURE 2:**
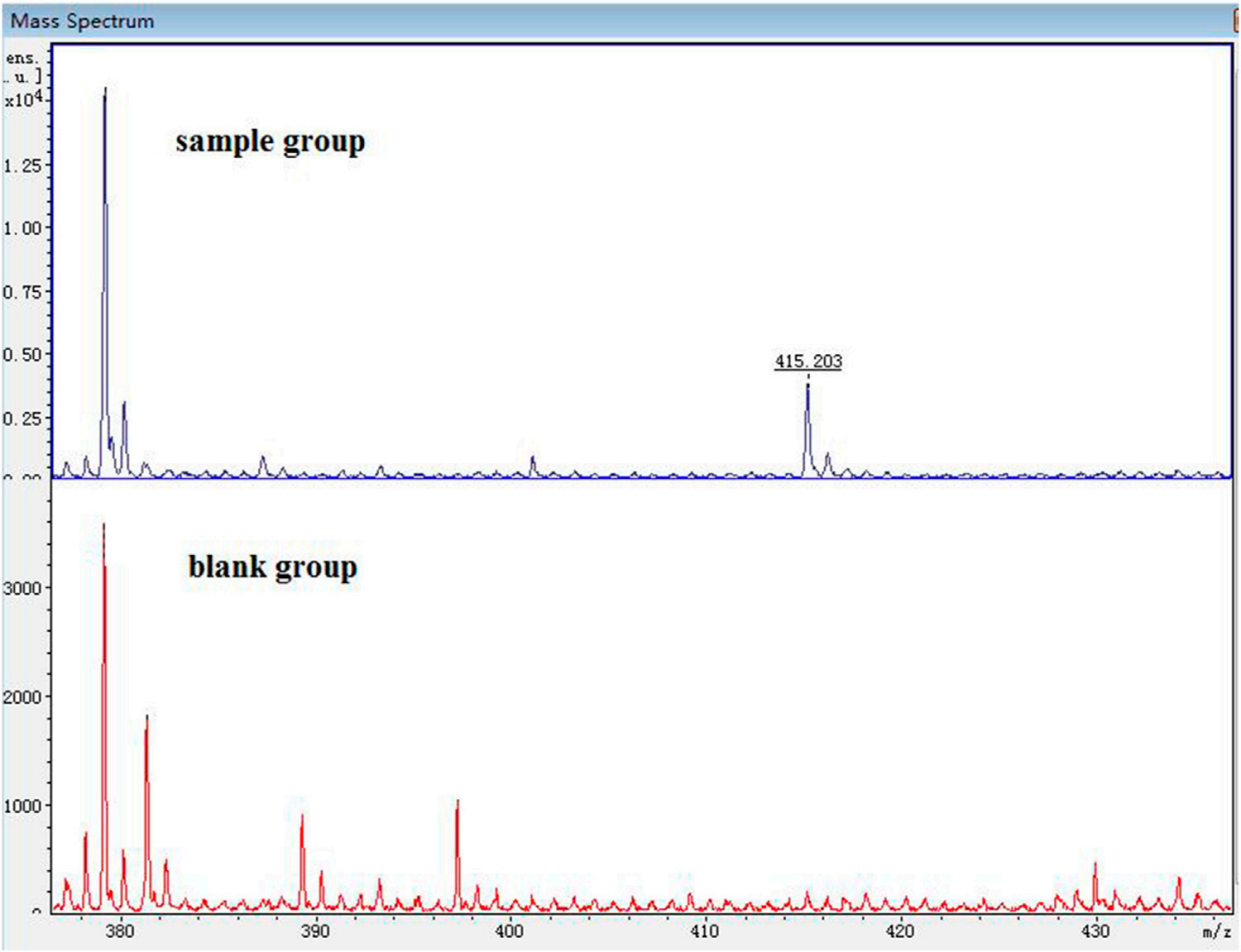
MALDI-TOF MS of the mice plasma dosed with D1 (sample group) or without D1 (blank group).

**FIGURE 3:**
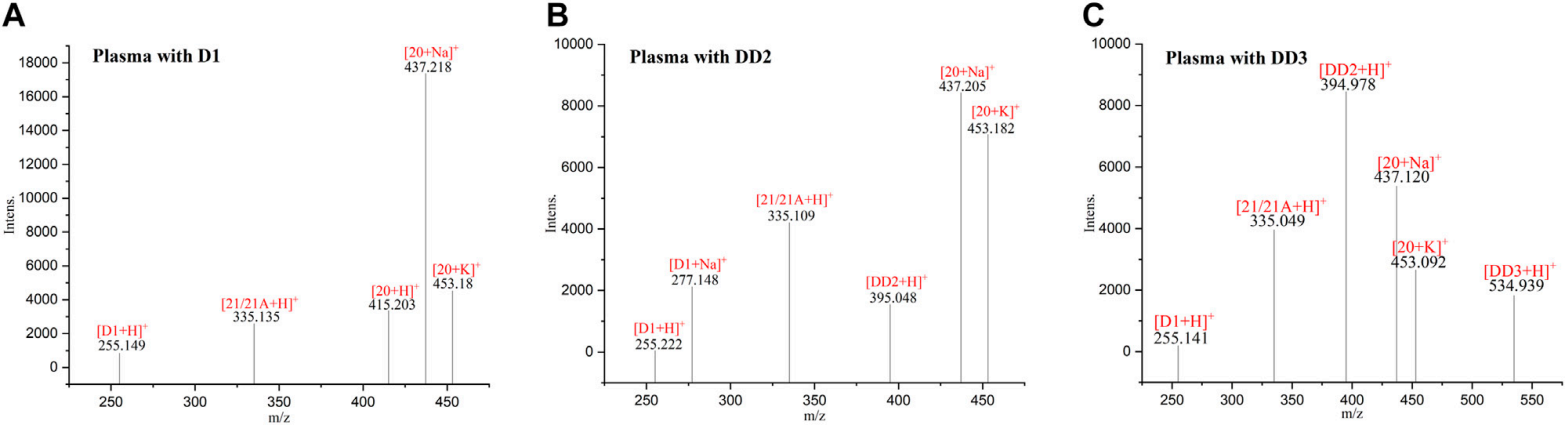
MALDI-TOF MS of the mice plasma dosed with D1 **(A)**, DD2 **(B)** and DD3 **(C)**.

The spectrogram of the blank and sample groups are shown in Figure 2 ([D1 bisulfate + H]^+^, m/z 415.203). The spectra of the other metabolites were obtained after subtracting the peak of the sample group from the peak of the blank group. The peaks of m/z 255.149 ([D1+H]^+^), 335.135 ([D1 monosulfate + H]^+^), 415.203 ([D1 bisulfate + H]^+^), 437.218 ([D1 bisulfate + Na]^+^), and 453.18 ([D1 bisulfonate + K]^+^) were observed in the plasma of the mice dosed with D1 (Figure 3) by comparing the spectra of the blank group and the control group. It was showed that the concentration of D1 was low, while the peak signals of its phase II metabolites, mono and disulfate, were strong. The peaks of m/z 255.222 ([D1+H]^+^), 277.148 ([D1+Na]^+^), 335.109 ([D1 monosulfate + H]^+^), 395.048 ([DD2+H]^+^),437.205 ([D1 bisulfate + Na]^+^), and 453.182 ([D1 bisulfate + K]^+^) were found in the plasma with DD2. The peaks of m/z 255.141 ([D1+H]^+^), m/z 335.049 ([D1 monosulfate + H]^+^), D1 monoester 394.978 ([DD2+H]^+^), m/z 437.120 ([D1 bisulfate + Na]^+^), and m/z 534.939 ([DD3+H]^+^) were found in the plasma with DD3. It was deduced that DD2 and DD3 were hydrolyzed to D1 *in vivo*, and the sulfation of D1 occurred, but D1 glucuronides were not found in the plasma. D1 can undergo both glucuronidation and sulfation in the plasma; however, it was a pair of competitive processes, and the sulfuric acid esterification occurred mainly at low concentrations (Peng et al., 2009). It was also possible that the glucuronide of D1 was easier to excrete into the urine via circulation (Shelnutt et al., 2000; Shelnutt et al., 2002).

### 3.3 MALDI-TOF MS of normal mice brain tissue sections

The peaks at 313.916 ([M14/17 + K]^+^), 335.937 ([M21/21A + H]^+^), and 437.038 ([D1 bisulfonate + Na]^+^) were found in the brain with D1. The peaks at m/z 275.291 ([M14/17 + H]^+^), which was supposed to be 3″-*OH*-*O*- DMA (14) or 5′-*OH*-*O*-DMA (17), and m/z 593.447 ([DD2 glucuronide + Na]^+^) were found in brain dosed with DD2 (Supplementary Figure S2). The phase I metabolite 14/17 is one of the terminal products of microbial degradation in the gastrointestinal tract of D1. It was indicated that prototype compounds may undergo the circulation of the liver and intestine and re-enter the circulatory system. The peak of m/z 335.257 ([D1 monosulfate + H]^+^) was found in the brain tissue after an intravenous administration of DD3. It was indicated that the metabolite daidzein sulfate could enter the brain through the blood–brain barrier and its phase I metabolites 14/17 also could enter the brain.

### 3.4 MALDI-TOF MS of normal mice heart tissue sections

The phase I metabolite peaks at m/z 259.517 ([M5+H]^+^) 297.517 ([M14/17 + Na]^+^), and 313.916 ([M14/17 + K]^+^) were found in the heart after the intravenous administration of D1 (Supplementary Figure S3, Scheme 1). The phase I metabolite 5 is *O*-desmethylangolensin (*O*-DMA) that is an end product of microbial degradation in the gastrointestinal tract of D1 (Frankenfeld, 2011), which is also suggested that the prototype compound D1 underwent the circulation of the liver and intestine. The phase II metabolite peak of m/z 571.486 ([DD2 glucuronide + H]^+^) was found in the heart with DD2, however, its phase II metabolites were not detected in the heart with DD3.

### 3.5 MALDI-TOF MS of normal mice kidney tissue sections

**FIGURE 4:**
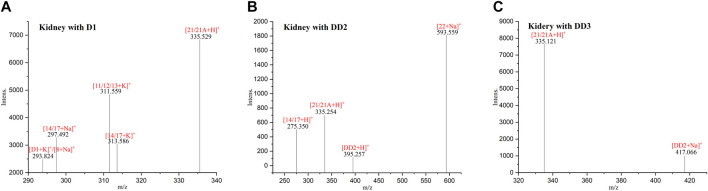
MALDI-TOF MS of the mice kidneys dosed with D1 **(A)**, DD2 **(B)**, and DD3 **(C)**.

The phase I metabolite peak of m/z 297.492 ([M14/17 + Na]^+^) and phase II metabolite peak of m/z 335.529 ([D1monosulfate + H]^+^) were found in the kidney with D1 (Figure 4). The phase II metabolite peaks of m/z 593.559 ([DD2 glucuronide + Na]^+^) and 335.254 ([D1monosulfate + H]^+^) were found in the kidney with DD2. And also, its prototype compound m/z 395.257 ([DD2+H]^+^) and its phase I metabolite peak of m/z 275.350 ([M14/17 + H]^+^) were found after an intravenous administration of DD2. The phase II metabolite peak at m/z 335.121 ([D1 monosulfate + H]^+^) and its hydrolysate 417.066 ([DD2+Na]^+^) were found after an intravenous administration of DD3.

### 3.6 MALDI-TOF MS of normal mice liver tissue sections

The phase I metabolite peak of m/z 313.576 ([M14/17 + K]^+^) and phase II metabolite peak of m/z 335.552 ([D1 monosulfate + H]^+^) were found in the liver after an intravenous administration of D1 (Supplementary Figure S4). The phase II metabolite peaks of m/z 593.276 ([DD2 glucuronide + Na]^+^) and 437.289 ([D1 bisulfate + Na]^+^) were found in the liver after an intravenous administration of DD2. The phase II metabolite peak of m/z 335.116 ([D1 monosulfate + H]^+^) was found in the liver after an intravenous administration of DD3.

### 3.7 MALDI-TOF MS of normal mice lung tissue sections

**FIGURE 5:**
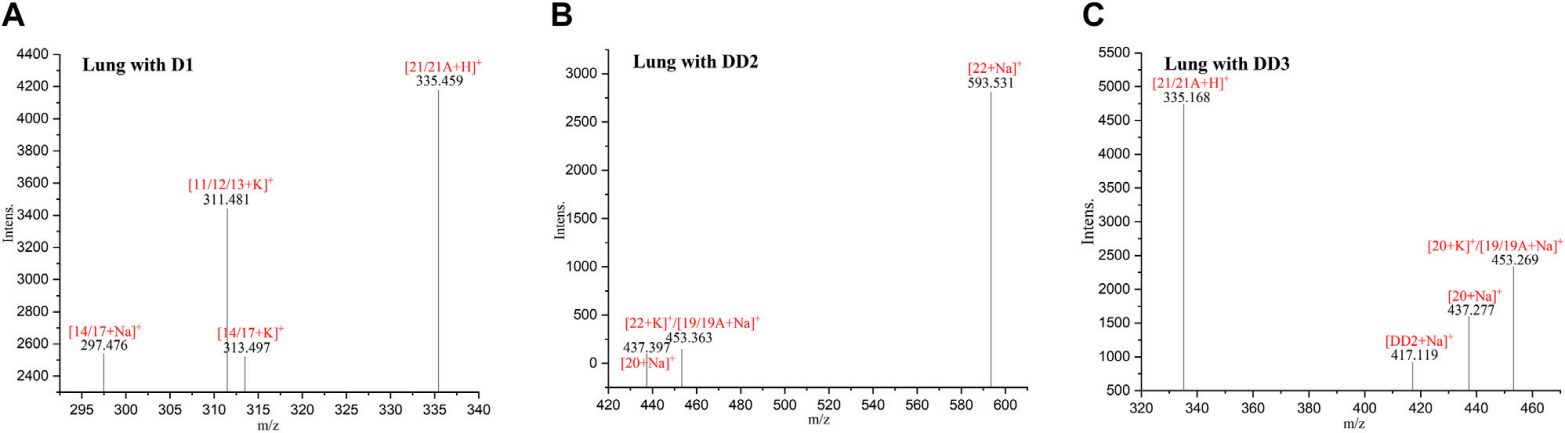
MALDI-TOF MS spectra of the mice lungs dosed with D1 **(A)**, DD2 **(B)**, and DD3 **(C)**.

The phase I metabolite peak of m/z 297.476 ([M14/17 + Na]^+^) and m/z 313,497 ([M14/17 + K]^+^) and phase II metabolite peak of m/z 335,459 ([D1 monosulfate + H]^+^) were found in the lungs after an intravenous administration of D1 (Figure 5). The phase II metabolite peak of m/z 437.397 ([D1 bisulfate + Na]^+^) and 453.363 ([D1 bisulfate + K]^+^) were found in the lungs after an intravenous administration of DD2. The phase II metabolite peak of m/z 335.168 ([D1 monosulfate + H]^+^), 437.277 ([D1 bisulfate + Na]^+^), 453.269 ([D1 bisulfate + K]^+^), and its hydrolysate 417.119 ([DD2+Na]^+^) were found in the lungs after the intravenous administration of DD3.

### 3.8 MALDI-TOF MS of normal mice spleen tissue sections

**FIGURE 6:**
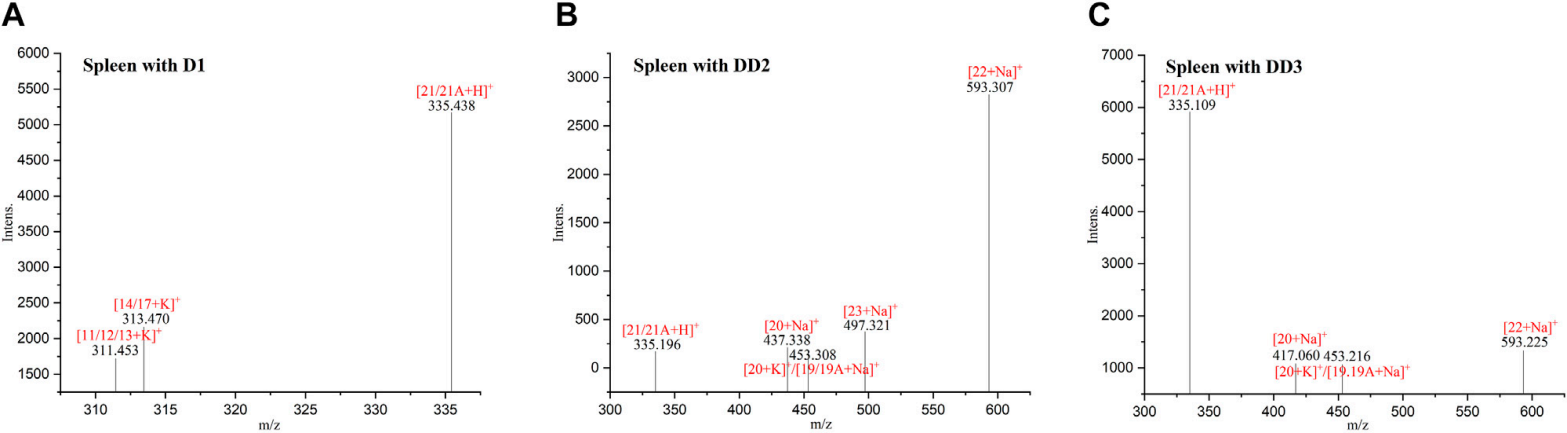
MALDI-TOF MS spectra of the mice spleens dosed with D1 **(A)**, DD2 **(B)**, and DD3 **(C)**.

The phase II metabolite peak of m/z 335.438 ([D1 monosulfate + H]^+^) was found in the spleen after the intravenous administration of D1 (Figure 6). The phase II metabolite peaks of m/z 335.196 ([D1 monosulfate + H]^+^), 437.338 ([D1 bisulfate + Na]^+^) and 453.308 ([D1 bisulfate + K]^+^) were found in the spleen after the intravenous administration of DD2. The phase II metabolite peaks of m/z 335.109 ([D1 monosulfate + H]^+^), 453.216 ([D1 bisulfate + K]^+^), and its hydrolysate 417.060 ([DD2+Na]^+^) were found in the spleen after the intravenous administration of DD3.

### 3.9 Mass spectrometry imaging

**FIGURE 7:**
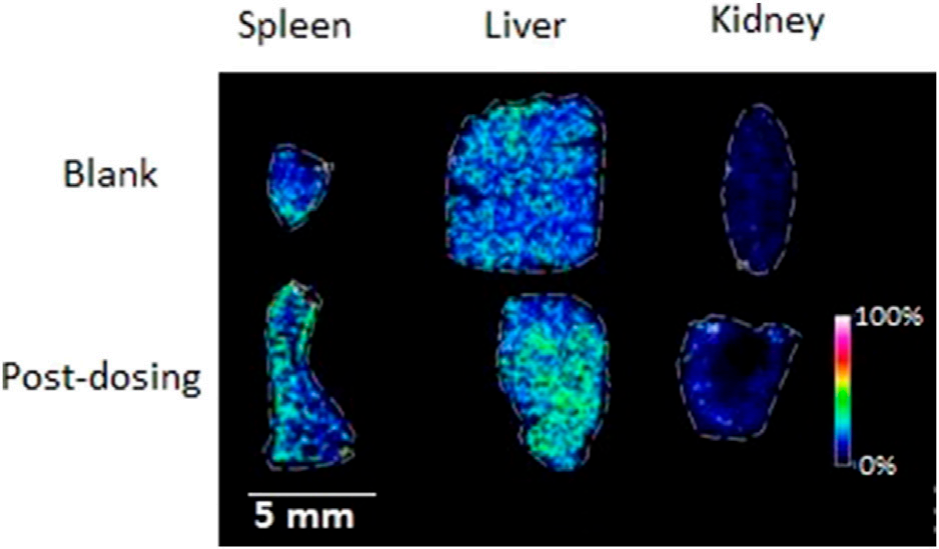
MSI of the mice organs dosed with (bottom) or without (top) DD2 (m/z 593 [DD2 glucuronide + Na]^+^).

The MALDI MSI was performed to get further observations of the specific distribution of the metabolites in the organs, sections of spleen, liver, and kidney. The compound DD2 contains the same function group as D1 (hydroxyl group) and DD3 (benzene sulfonyl group). In addition, the anti-inflammatory and other activities of DD2 (Ref. 2) were the highest compared to D1and DD3. Therefore, DD2 was selected for further mass spectrometry imaging to investigate the specific distribution of its metabolites in the organs. The highlighted portion in the MSI represented DD2 sodium glyconate. The brightness of the sample group was macroscopically significantly different from that of the blank group as shown in MSI. The MSI showed that the DD2 sodium glyconates (m/z 593) were distributed in the liver and kidney evidently compared with the blank organs, and it is evenly distributed in the liver but is mainly distributed in the marginal zone in the kidney (Figure 7). The results reported by Redmon (Redmon et al., 2016) showed that glucuronidation was the main metabolic pathway of D1 in mouse liver microsomes. Therefore, it was inferred that glucuronidation of its derivative DD2 occurred primarily in the liver. And, glucuronidation entered the spleen and kidneys through enterohepatic circulation. DD2 sodium glyconate was largely concentrated in the liver after 4 h of injection. It indicated that glucuronidation of this class of compounds was the main metabolic way *in vivo*. Combined with the data in Figure 9, it could be seen that, DD2 glucuronide was similarly widespread in the lungs and heart, and also entered the brain through the blood–brain barrier.

### 3.10 Molecular docking results

**FIGURE 8:**
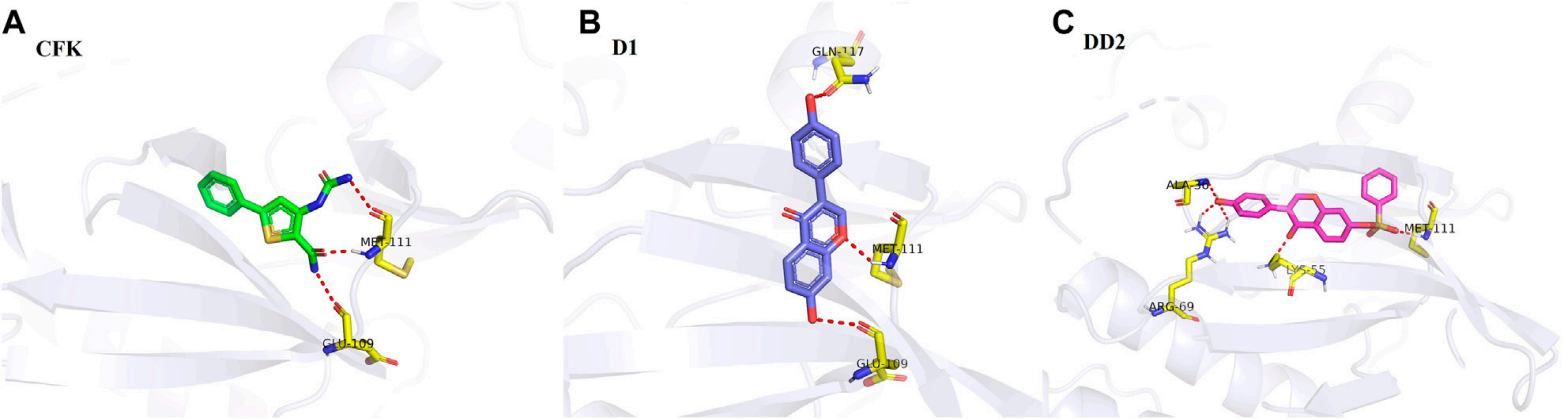
Visual results of the docking between ligands and proteins.

The molecular docking visualization analysis was performed using the PyMOL software to compare the ligands with higher docking scores and more stable conformations. The binding mode of the target protein receptor to the ligand and the interactions with the surrounding amino acid residues are shown in Figure 8. The re-docking results of CFK with JNK1 were consistent with the data obtained in the literature (Oza et al., 2012). CFK was combined with MET111 and GLU109 to form 3 hydrogen bonds (Figure 8A). D1 was combined with MET111, GLU109, and GLN117 to produce 3 hydrogen bonds (Figure 8B). While DD2 was combined with MET111, LYS55, ARG69, and ALA36 to generate 5 hydrogen bonds (Figure 8C).

The binding activity and possibility were determined according to the binding energy of the ligand and receptor. The binding energy results statistics are shown in Table 1. The binding energy obtained from the re-docking between CFK and JNK1 was −6.65 kcal/mol with an inhibition constant of 13.41 μmol/L. The docking of D1 to JNK1 yielded a binding energy of −7.04 kcal/mol and an inhibition constant of 6.88 μmol/L. The binding energy of DD2 and JNK1 is as low as −9.5 kcal/mol, and the inhibition constant is 0.1 μmol/L.

**TABLE 1:** Docking results of the ligands and JNK1.

Entry	Binding energy (kcal/mol)	H-bonds	Inhibitor constant (μmol/L)
CFK	−6.65	3	13.41
D1	−7.04	3	6.88
DD2	−9.5	5	0.1

## 4 Discussion

**SCHEME 2:**
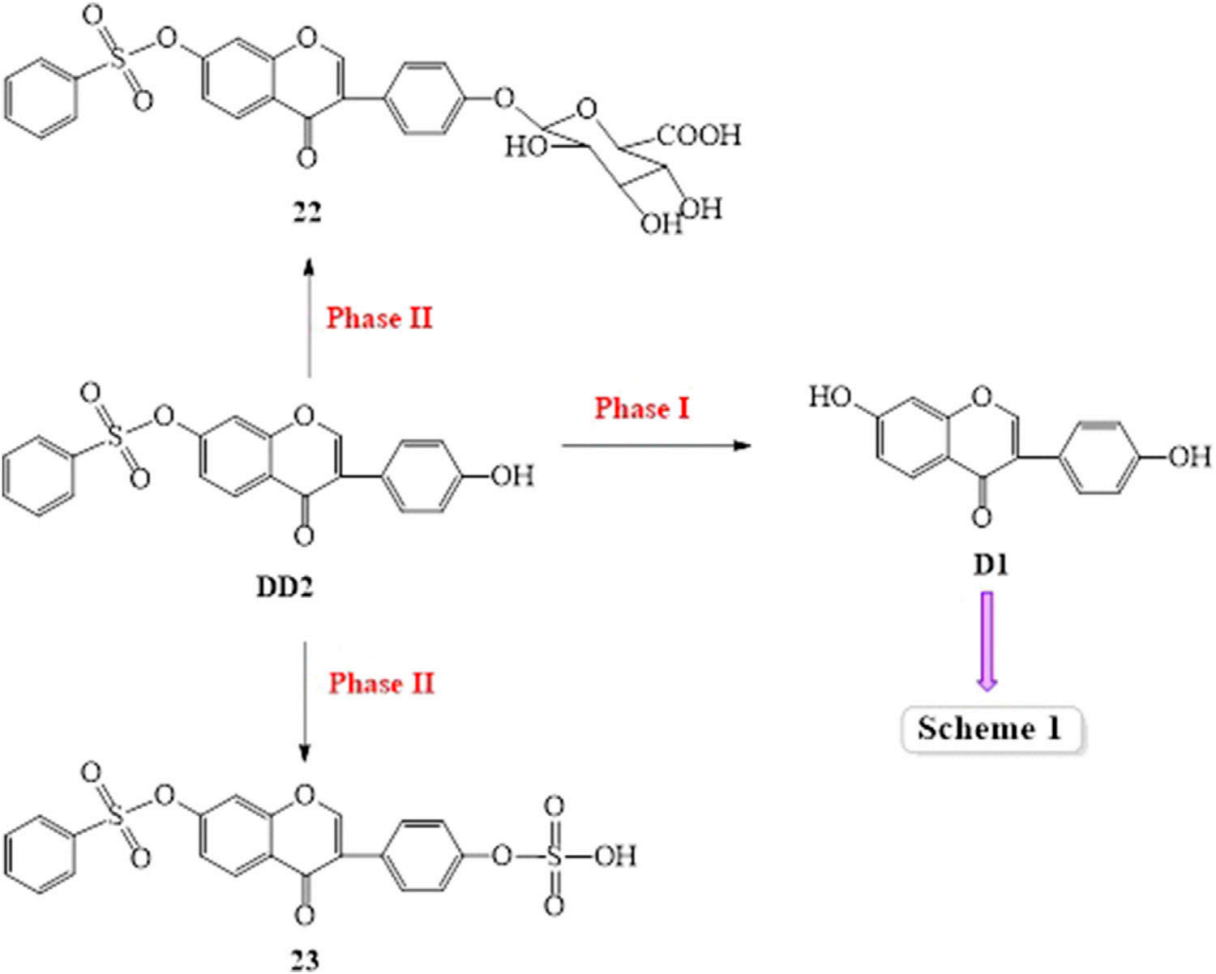
Possible metabolic pathways of DD2

**SCHEME 3:**
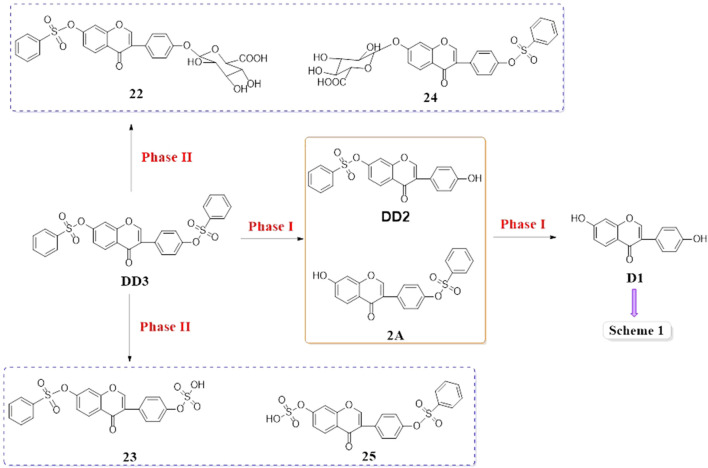
Possible metabolic pathways of DD3

Based on the results of the existing studies, it may be concluded that D1 was absorbed into the plasma, metabolized by a combination of hydroxyl groups with glucuronic acid or sulfuric acid, and partially excreted in its original form. On the basis of previous literature studies, the possible metabolic pathways of DD2 and DD3 are shown in Scheme 2 and Scheme 3.

**FIGURE 9:**
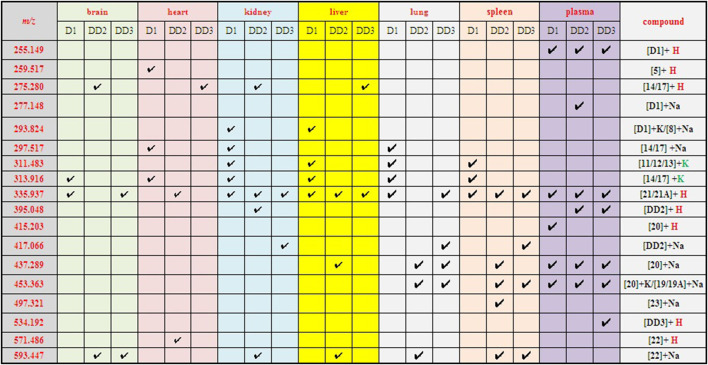
The metabolites of D1, DD2, and DD3 in mice.

The prototype compound D1 was found in the plasma after the intravenous administration of D1. In the meantime, daidzein monosulfate (21/21A) was found in the plasma, liver, kidneys, lungs, and spleen, and daidzein bisulfate compound (20) was found in the plasma and brain. The O-DMA (5) was found in the heart which indicated that twice reductive hydrogenation of D1 with reductases produced O-DMA *in vivo*, 3′-OH-D1, 6-OH-D1, or 8-OH-D1 was also formed via hydroxylation of D1 and then 3′-OH-O-DMA (14) or 5-OH-O-DMA (17) was obtained by twice reductive hydrogenation in the liver, kidney, and lung (Scheme 1, Figure 9). It was indicated that D1 may undergo the circulation of the liver and intestine and re-enter the circulatory system after it entered the liver via the caudal vein. The glucuronide product of D1 was not found under the same condition. The possible reason was that the concentration of D1 *in vivo* is too low. The glucuronide and sulfate of D1 is a pair of competitive metabolic reactions. When the concentration of D1 is high, daidzein glucuronide conjugation is the main process, but sulfuric acid esterification of D1 is the main one with low concentration of D1.

D1 was found in the plasma after the intravenous administration of DD2. It was indicated that the hydrolysis of DD2 to form D1 by esterase *in vivo*. Simultaneously, the DD2 glucuronide was also found in the liver and kidney, indicating that the glucuronide conjugation metabolism occurred *in vivo*. The other metabolisms were similar to those of D1 (Scheme 1, Scheme 2). DD2 or its isomer 2A and D1 were found in the plasma except for the prototype compound DD3 after the intravenous administration of DD3, suggesting that it was hydrolyzed *in vivo* (Scheme 3). Further metabolisms took place after the hydrolysis of DD2 or 2A to D1. Metabolites DD2 or 2A appeared in the kidney, lung, and spleen (Figure 9).

It is considered that the ligand and protein have a good binding ability when the binding energy is <5 cal/mol (Li et al., 2019). It was concluded that the docking model was credible from the docking results of CFK with JNK1. It is generally believed that the lower the binding energy of a ligand to a receptor is, the more stable its bound conformation is (Liu et al., 2020). The binding energies of DD2 and D1 to their target proteins are less than CFK. It indicated that D1 and DD2 could be potentially good JNK inhibitors. At the same time, the number of hydrogen bonds formed by DD2 and the protein was more than that of D1 and CFK. It also explained that the anti-inflammatory activity of DD2 was much greater than that of D1 in our previous report (Peng et al., 2017).

## 5 Conclusion

In this study, a new strategy was established to investigate the distribution and metabolism of daidzein and its benzene sulfonates *in vivo* (in mice) based on MALDI-TOF MSI. To the best of our knowledge, it is the first to report on the distribution and metabolism of D1 and its derivatives in the various organs of mice. Molecular docking results shed light on the mechanism of the anti-inflammatory activity of D1 and its derivatives at the protein level. It will provide beneficial guidance for further pharmacological, toxicological studies and the clinical-use research of these compounds.

## Data Availability

The datasets presented in this study can be found in online repositories. The names of the repository/repositories and accession number(s) can be found in the article/Supplementary Material.
