# Supplementary figures and images for: Distribution and metabolism of daidzein and its benzene sulfonates in vivo (in mice) based on MALDI-TOF MSI
Source: Front Pharmacol. 2022 Aug 10;13:918087. doi: 10.3389/fphar.2022.918087 (PMC9399426; doi:10.3389/fphar.2022.918087)

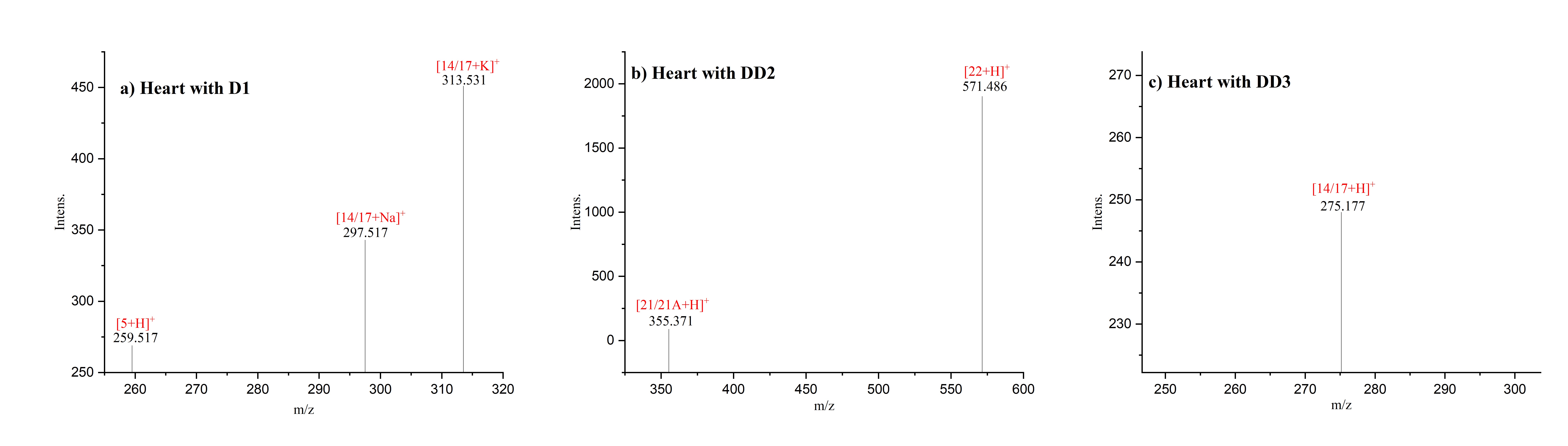

Supplement: Supplementary file 1 [file Image3.JPEG]

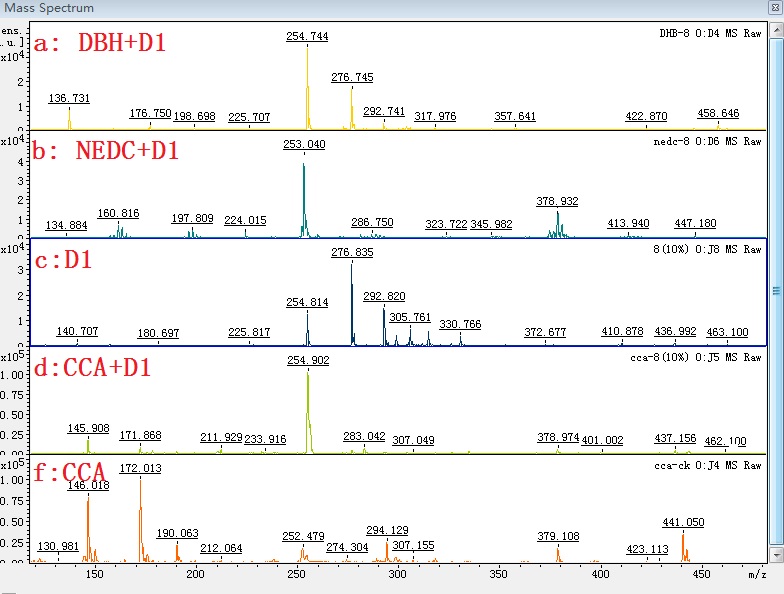

Supplement: Supplementary file 2 [file Image1.JPEG]

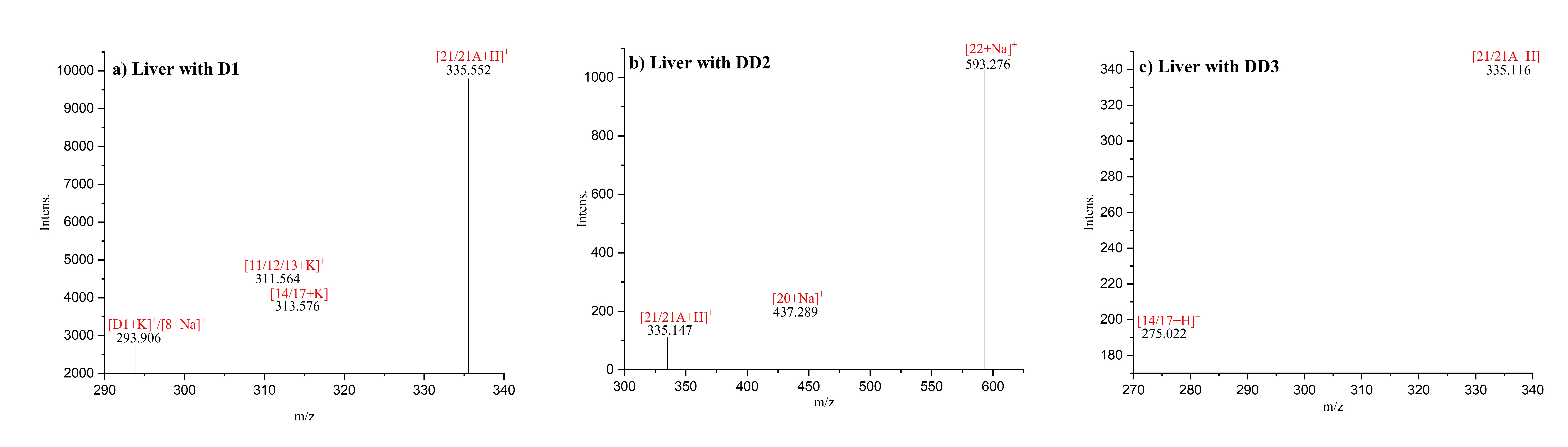

Supplement: Supplementary file 3 [file Image4.JPEG]

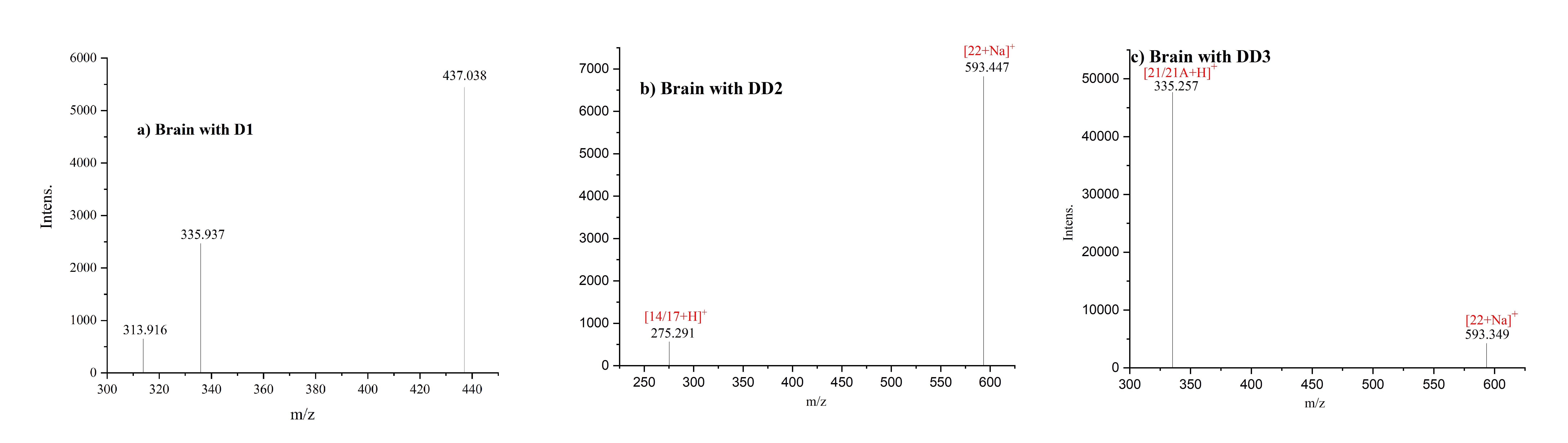

Supplement: Supplementary file 4 [file Image2.JPEG]
